# Correction and removal of expression of concern: Potential of non-thermal discharge plasmas for activated sludge settling: effects and underlying mechanisms

**DOI:** 10.1039/d6ra90089e

**Published:** 2026-08-07

**Authors:** Yun Chen, Siqi Liu, Zhiyin Ren, Qi Wang, Ying Zhang, Yajie Zuo, Jian Zhou, Hongtao Jia, Tiecheng Wang

**Affiliations:** a Ningxia Houde Environmental Protection Technology Co., Ltd Yinchuan 750000 China; b College of Information Science and Technology, Nanjing Forestry University Nanjing 210037 China; c College of Natural Resources and Environment, Northwest A&F University Yangling Shaanxi Province 712100 PR China wangtiecheng2008@126.com; d Key Laboratory of Plant Nutrition and the Agri-Environment in Northwest China, Ministry of Agriculture Yangling Shaanxi 712100 PR China; e College of Resources and Environment, Xinjiang Agricultural University Urumqi 830052 China

## Abstract

Correction and removal of expression of concern for ‘Potential of non-thermal discharge plasmas for activated sludge settling: effects and underlying mechanisms’ by Yun Chen *et al.*, *RSC Adv.*, 2023, **13**, 19869–19880, https://doi.org/10.1039/D3RA02921B.

The authors regret errors in Fig. 6, 9b and 10.

Northwest A&F University have confirmed the integrity and the reliability of the results, and recommended the publication of a correction.

The SEM data in Fig. 6 were not collected under the described conditions. The experiments have been repeated and the corrected Fig. 6 is shown below.

**Fig. 1:**
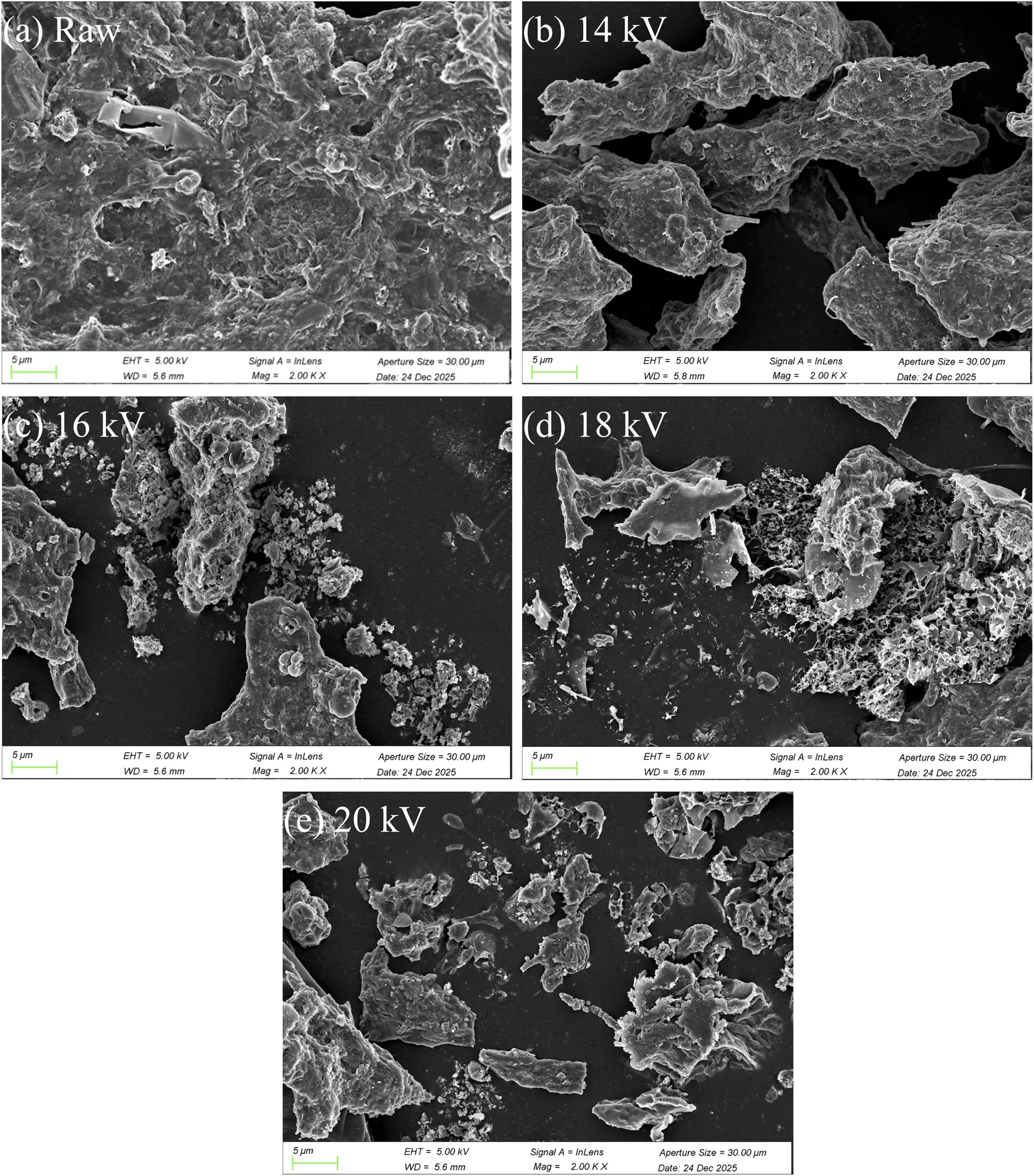
SEM images of sludge after 60 minutes of discharge plasma treatment at different voltages: (a) raw sludge (0 kV); (b) 14 kV; (c) 16 kV; (d) 18 kV; (e) 20 kV. The raw sludge shows compact, large floccular aggregates. With increasing voltage, the structure disintegrates into smaller fragments and fine particles, indicating the disruption of the floc matrix by plasma-generated reactive species.

Accordingly, the paragraph beginning “Fig. 6 shows the SEM images…” should be revised to: “Fig. 6 shows the SEM images of the sludge after 60 minutes of discharge treatment at various voltages. The raw sludge before treatment showed compact, large floccular aggregates. With increasing voltage, the structure disintegrated into smaller fragments and fine particles, indicating the disruption of the floc matrix by plasma-generated reactive species. The strong oxidizing reactive oxygen species produced in the discharge plasma system destroyed the microbial cells and the massive floc structure of the sludge, promoting the release of organic matter and water inside the floc from the mud phase to the liquid phase. The reactive oxygen species also destroyed extracellular polymeric substances wrapped in the floc surface, resulting in a rough floc surface”.

When conducting further EPR analysis, the authors found that there was no detectable ˙O_2_^−^ signal in the system when sludge was present. Fig. 9b has been corrected and is shown below.

**Fig. 2:**
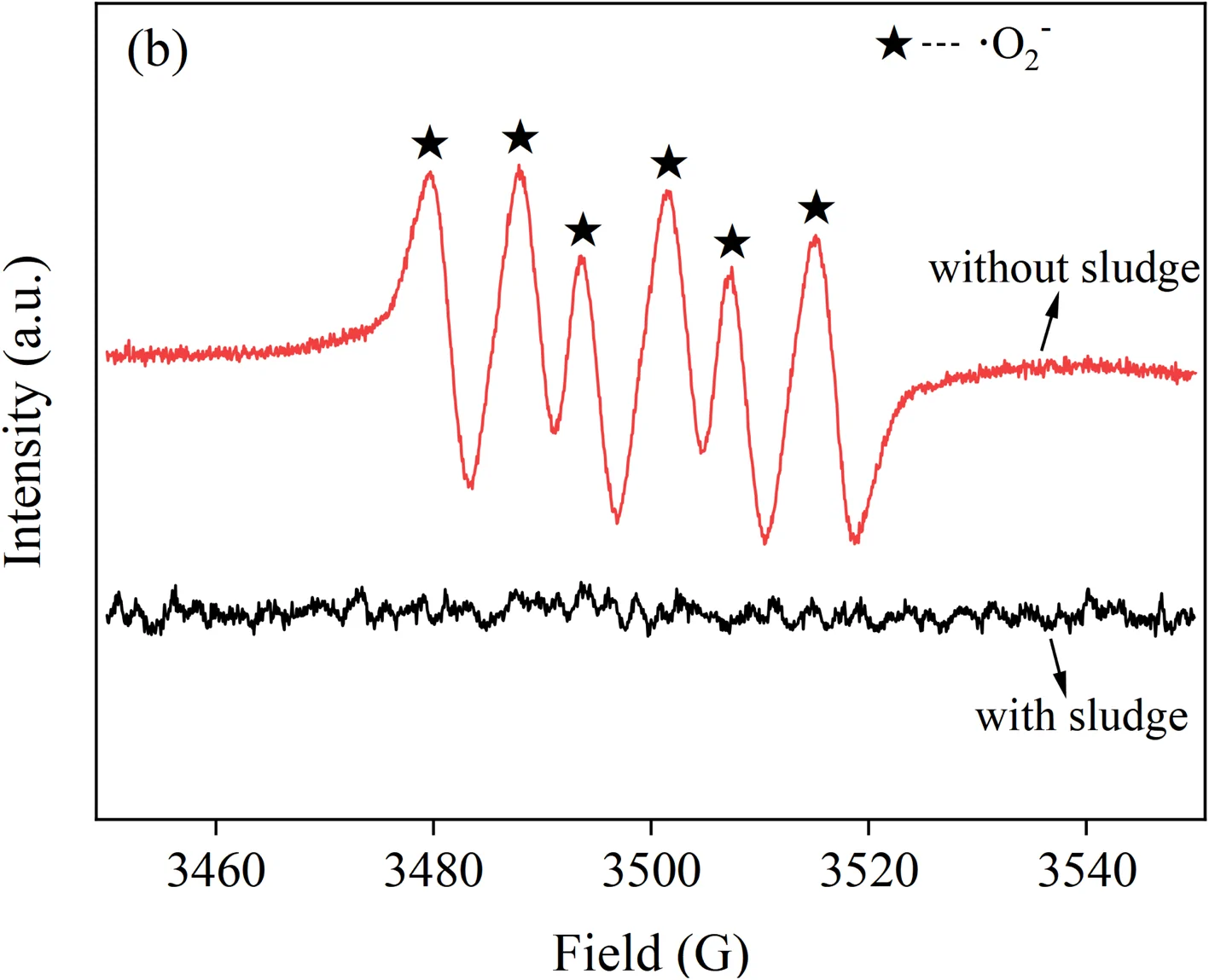
(b) EPR spectra of superoxide radicals (˙O_2_^−^) detected in the plasma system. The spectra labeled “without sludge” were obtained in pure water, while “with sludge” represent signals detected immediately upon interaction with the sludge mixture. Note: the signal for ˙O_2_^−^ in the presence of sludge is absent, indicating its rapid consumption in reactions with sludge components.

The corrected version of Fig. 9b provides support for the proposed reaction mechanism by further confirming the rapid consumption of ˙O_2_^−^ as a transient intermediate during the treatment process.

The sentence beginning “The contributions of ˙OH and O_2_˙^−^ were also confirmed…” should be revised to: “Notably, the signal for ˙O_2_^−^ was absent in the presence of sludge, indicating its rapid consumption in reactions with sludge components, which is consistent with the corrected Fig. 9b”.

The experiment in Fig. 10 has been repeated to include standard deviation and statistical significance test results (*p* < 0.05) and the corrected Fig. 10 is shown below.

**Fig. 3:**
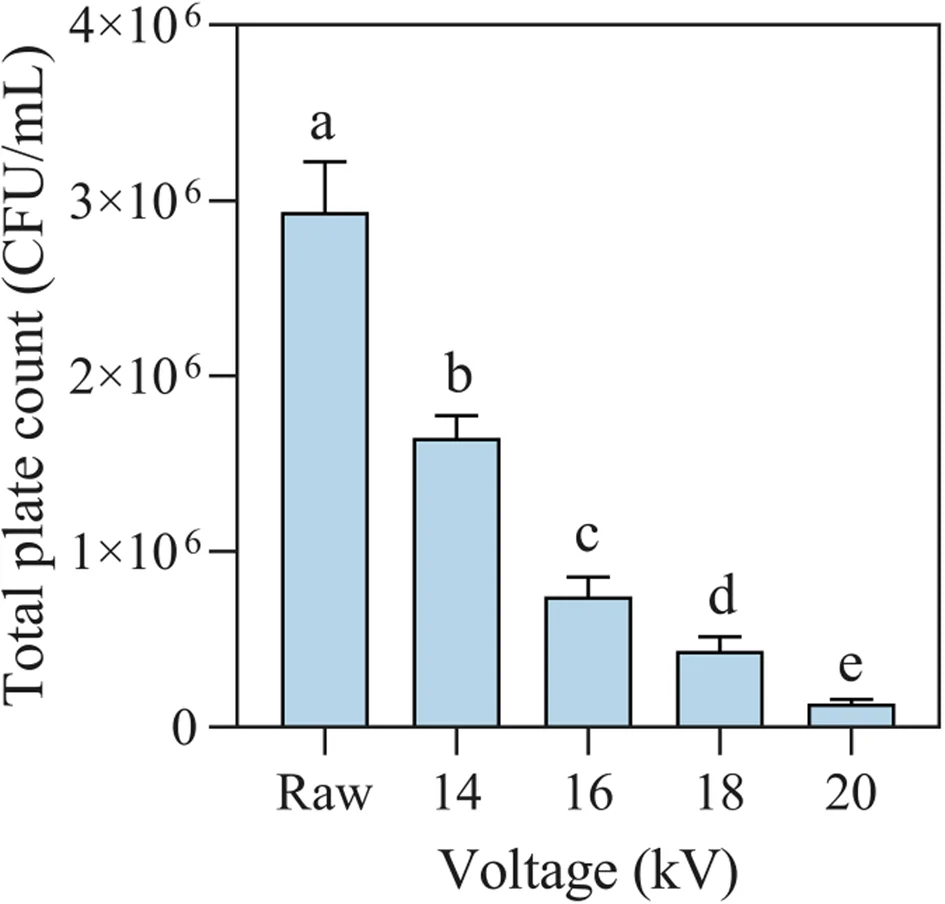
Total cultivable bacterial count in the sludge mixture after 60 minutes of treatment with discharge plasma at different voltages. Data are presented as mean ± standard deviation (*n* = 3). Different lowercase letters (a to e) above the bars indicate statistically significant differences among groups (*p* < 0.05, one way ANOVA followed by Tukey's test).

The sentence beginning “Fig. 10 shows the influence of…” should be revised to “Fig. 10 shows the influence of discharge voltage on the number of total cultivable bacteria after 60 min treatment”.

To accurately reflect the measured parameters, all instances of “*E. coli*” in the main text should be corrected to “total cultivable bacteria” to maintain consistency with the revised figure caption.

Readers are advised to refer to the corrected figures published in the correction notice.

This notice supersedes the information provided in the Expression of Concern related to this article.

The Royal Society of Chemistry apologises for these errors and any consequent inconvenience to authors and readers.

